# Low-dose oral cholecalciferol is associated with higher numbers of Helios^+^ and total Tregs than oral calcitriol in renal allograft recipients: an observational study

**DOI:** 10.1186/s40360-016-0066-9

**Published:** 2016-06-14

**Authors:** Mostafa G. Aly, Karina Trojan, Rolf Weimer, Christian Morath, Gerhard Opelz, Mohammed A. Tohamy, Volker Daniel

**Affiliations:** Transplantation-Immunology, Institute of Immunology, University Hospital Heidelberg, Im Neuenheimer Feld 305, 69120 Heidelberg, Germany; Department of Nephrology, University of Heidelberg, Heidelberg, Germany; Department of Internal Medicine, University of Giessen, Klinikstrasse 33, D-35385 Giessen, Germany; Nephrology Unit, Internal Medicine Department, Assiut University, Assiut, Egypt

**Keywords:** Treg, Cholecalciferol, Calcitriol, Renal transplantation

## Abstract

**Background:**

Regulatory T cells (Tregs) are a cornerstone of graft acceptance. High numbers of Tregs are associated with better long-term graft survival. Recently, Vitamin D was suggested as an immunomodulator, in addition to its classical role in calcium metabolism. Vitamin D modulates Tregs and might, thereby, promote graft acceptance and long-term graft survival.

**Methods:**

One hundred twenty-three renal allograft recipients attending either Heidelberg nephrology or Giessen internal medicine clinic were enrolled in this cross- sectional study. Sixteen healthy controls were studied in addition. Sixty-nine patients were receiving no vitamin D, 38 calcitriol, and 16 cholecalciferol supplementations. We evaluated whether there was a difference in the absolute numbers of Helios^+^, Helios^−^, CTLA-4^+^, IFNg^+^, and total Tregs among the patient groups.

**Results:**

Cholecalciferol supplementation was associated with higher absolute numbers of Helios^+^, CTLA-4^+^, and total Tregs than calcitriol (*p* < 0.001, *p* = 0.004, *p* = 0.001 respectively). Helios^+^ Tregs were also higher in cholecalciferol than no vitamin D supplementation patients (*p* = 0.001), whereas CTLA-4^+^ and total Tregs were similar in both groups (*p* = NS). Helios^+^, Helios^−^, CTLA-4^+^, IFNg^+^, and total Tregs were similar in the cholecalciferol and healthy control groups (*p* = NS).

**Conclusion:**

Our findings indicate that cholecalciferol, even when administered at low dosages, has a stabilizing effect on Tregs (particularly the Helios + subset), in contrast to calcitriol which showed neither a stabilizing nor a proliferation-inducing effect on the same cell population.

## Background

T regulatory cells (Tregs) represent a subset of professional cells with powerful immunosuppressive activity. They modulate the immune responses, abrogate autoimmune diseases, and maintain self-tolerance. High numbers of Tregs in peripheral blood of renal transplant recipients were shown to be associated with long-term renal graft survival [1]. Generally, Tregs express the surface markers CD4^+^ CD25^+^ Foxp3^+^ CD127 ^low/-^ and can be divided into three distinct subtypes according to their origin: Thymus-derived Tregs (tTregs), peripherally induced Tregs (pTregs), and in vitro-induced Tregs (iTregs) [2]. tTregs originate in the thymus and usually express Helios; a member of the Ikaros family transcription factor. pTregs are induced in the periphery upon exposure to antigens in the absence of inflammatory cytokines. pTregs are antigen specific, express unstable Foxp3, and are often Helios negative [3–5]. The exact function of Helios remains unclear, however, Getnet et al. showed that Helios enhanced Foxp3 expression [6]. Whether Helios^+^ and Helios^−^ Tregs have similar suppressive capacity is uncertain. Whereas Himmel et al. reported that both types had comparable immunosuppressive capacity, [4] Elkord et al. demonstrated that Helios^+^ Tregs possessed more suppressive capacity in vitro [7]. Zabransky et al. reported that the in vitro suppressive function of Tregs correlated with the absolute numbers of Helios^+^ cells. [8] Accordingly, it seems likely that Helios^+^ Tregs represent a highly suppressive Treg subset. Recently, a new Treg subset, Interferon-gamma producing Tregs (IFNg^+^ Tregs), was discovered in mice and humans [9, 10]. These cells represent the first line of Tregs and exert a suppressive effect on an initial immune response. They include tTregs as well as pTregs [11]. Among many other surface receptors, Tregs express cytotoxic T-lymphocyte-associated protein 4 (CTLA-4), which is involved in cell-cell inhibition. Accordingly, CTLA-4 maintains the suppressive capacity of Tregs in animal models [12].

Beyond its role in calcium homeostasis, vitamin D plays an important role as an immunomodulator. Through the interaction of vitamin D with its intracellular receptor (VDR), and subsequently with the vitamin A/ vitamin A receptor complex, it enters the nucleus, binds to the vitamin D response elements (VDRE) of the promoter regions of different genes, and ultimately modifies the transcription of more than 900 genes [13]. Thereby, vitamin D can affect, either directly or indirectly, about 3 % of the human genome [14–16]. Ardalan et al. showed that when calcitriol was administered to the donor before renal transplantation, and was continued in the recipients thereafter, it led to a significant increase in the number of CD3^+^CD4^+^CD25^+^ cells [17]. A complicating finding is that the resulting higher incidence of hypercalcemia renders the administration of large doses of calcitriol potentially toxic. In contrast, cholecalciferol (vitamin D_3_) is rather safer when administered in large doses [18]. Cholecalciferol, the native form of vitamin D, undergoes two hydroxylations: the first in the liver to 25 (OH) D_3_ (calcidiol), and the second in the kidney to form calcitriol. While administration of a weekly large dose of oral cholecalciferol (140,000 IU/month) for 3 months was reported to significantly increase the numbers of peripheral Tregs in vivo in healthy individuals and patients with Type 1 diabetes [19, 20], this was challenged by Smolders et al., who demonstrated that supplementation with an oral daily dose of 20,000 IU of cholecalciferol for 3 months in relapsing remitting multiple sclerosis patients did not significantly increase the numbers or the suppressive action of Tregs [21].

Recently, in vitro experiments demonstrated that activated T cells express 1α-hydroxylase and have the capacity to convert 25(OH)D_3_ to 1,25(OH)_2_D_3_ in sufficient concentrations to affect the vitamin D- responsive genes [22]. Interestingly, the numbers of Foxp3^+^ Tregs and the expression of CTLA-4 were increased after stimulation of CD4^+^CD25^−^ cells only in the presence of dendritic cells and 25(OH) D_3_, in contrast to 1,25(OH)_2_ D_3,_ which in supra-physiological concentrations increased the numbers of Foxp3^+^ Tregs and expression of CTLA-4, regardless of the presence of dendritic cells [23]. Thus, in vitro, calcitriol can induce Tregs without the presence of dendritic cells.

In vivo studies on effects of cholecalciferol and calcitriol on different subsets of Tregs in renal allograft patients are scarce. The available publications on in vivo and in vitro studies do not explicitly show which vitamin D form was associated with significantly higher numbers of Tregs in renal allograft patients. In the present study, we were interested in investigating whether the administration of either form of vitamin D is associated with a preferential increase in the numbers of certain subsets of Tregs. From a clinical perspective, it is important to determine whether supplementation with one form or the other in the usual doses prescribed for post transplantation calcium homeostasis is superior regarding an achievement of higher numbers and better suppressive capacity of Tregs. To the best of our knowledge, we believe this is the first study to compare the association of two vitamin D forms with the numbers of Helios^+^, Helios^−^, and CTLA-4^+^ Tregs in renal allograft recipients.

## Methods

### Patients

This cross-sectional study was conducted between April 2014 and July 2015. Blood samples were collected from renal transplant patients attending outpatient clinics at Heidelberg nephrology and Giessen internal medicine departments. All the samples were analyzed by the same operator at the transplantation immunology department of Heidelberg University. The numbers of Helios^+^, Helios^−^, CTLA-4^+^, IFNg^+^, and total Tregs were analyzed in 123 renal transplant patients. As an ancillary part, we analyzed retrospectively whether the patients were supplemented with vitamin D forms or not for at least six months before the time of blood sampling for the T cell subpopulation studies. Patients who received post-transplant vitamin D supplementations for shorter than 6 months before blood sampling were excluded from the study. Fifty-four patients were supplemented post transplantation with vitamin D forms, of whom 38 received oral calcitriol and 16 oral cholecalciferol. Sixty-nine patients were not supplemented. The three groups were compared with 16 healthy controls studied in parallel. The healthy controls were lab staff members who were not suffering from any acute infections or chronic diseases, and were not taking any medications. The patients were reasonably matched regarding gender, age, and follow up time post renal transplantation (*P* = NS), however, they differed from the healthy controls regarding gender and age (Table 1).

**Table 1:** Demographic and baseline characteristics of 139 study patients according to vitamin D- supplementation status and form (Non-vitamin D, calcitriol, or cholecalciferol)

	Controls	Non-vitamin D	Calcitriol	Cholecalciferol	*P* value
No. of subjects	16	69	38	16	
Gender (% female)	81	35	29	43	0.003
Age (years)	42 (25.5–53.5)	53 (41.2–62.7)	57 (49–71)	55 (45.5–64)	0.004
Years post transplantation		2.1 (1.3–5.6)	3 (1.7–6.2)	1.5 (1.1–2.4)	0.117
Vitamin D weekly dose					
Calcitriol (μg)			1.75 (1.75–3.5)		
Cholecalciferol (IU)				7000 (5950–10,000)	
No. of rejections		5	1	2	0.275
CMV IgG positive recipients %		52.5	58.1	56.3	0.894
ATG induction (% of patients)		13	17	23	0.568
Serum Creatinine mg/dl		1.5 (1.2–1.9)	1.6 (1.3–2.2)	1.2 (1.1–1.7)	0.155
Maintenance immunosuppresion (% of patients)					
Tac/CsA+ MPA/MMF + Steroids		84	72	76	0.272
Tac/CsA+ AZA + Steroids		13	9	12	
Tac/CsA+ everolimus/sirolimus + Steroids		3	19	12	
C_0_ MPA (mg/l)		1.9 (1.2–4)	2.6 (1.7–4)	2.5 (1.7–5.2)	0.293
C_0_ Tac (ng/ml)		6 (5.2–7.8)	6.6 (5.7–7.3)	6.3 (4.8–7.3)	0.839
C_0_ CycA (ng/ml)		116 (93–128)	140 (117–169)	63^a^	
Time of blood sampling (% of patients)					
Summer	25	45	52.6	31.25	
Autumn	25	29	21.1	56.25	0.252
Winter	25	13	15.8	6.25	
Spring	25	13	10.5	6.25	
NK cells/μl	176 (149–239)	141 (75–191)	106 (43–230)	178 (90–296)	0.084
CD8^+^ T cells/μl	377 (291–556)	310 (200–544)	417 (282–568)	531 (237–568)	0.439
CD4^+^ T cells/μl	762 (590–990)	677 (373–1043)	524 (357–877)	595 (352–1070)	0.144
Tregs/μl*	5.5 (4.7–8.4)	3.1 (1.6–5.3)	2.2 (1.3–4.3)	4.7 (3–8.5)	<0.001
Helios^+^ Tregs/μl*	2.5 (1.3–4)	0.7 (0.3–1.4)	0.6 (0.3–1.1)	2.2 (1.1–3.8)	< 0.001
Helios^−^ Tregs/μl*	3.3 (2.5–4.2)	2.2 (0.9–3.9)	1.4 (0.9–3.1)	2.7 (1.1–4.6)	0.008
IFNg^+^ Tregs/μl*	0.3 (0.1–0.7)	0.3 (.04–0.3)	0.16 (0.04–0.4)	0.2 (0.1–0.7)	0.026
CTLA-4^+^ Tregs/μl*	1.5 (1–2.8)	0.4 (0.2–0.9)	0.35 (0–0.9)	1.2 (0.2–3.5)	< 0.001
Treg/CD4^+^ T cells %	0.72 (0.63–0.91)	0.46 (0.3–0.79)	0.42 (0.26–0.89)	0.79 (0.53–1.4)	< 0.001
Helios^+^ /Helios^−^ Tregs	0.72 (0.41–1.1)	0.38 (0.27–0.61)	0.34 (0.21–0.65)	0.65 (0.39–1.4)	0.011
Treg/CD8^+^ T cells %	1.4 (0.94–2.4)	0.8 (0.4–1.5)	0.7 (0.3–1.1)	1.4 (0.9–1.8)	0.002

Data are presented as median + interquartile range. Asterisks refer to Tregs with the marker combination CD4^+^ CD25^+^ CD127^−^ Foxp3^+^

*NK* natural killer cells, *CMV* cytomegalovirus, *ATG* Anti-thymocyte globulin, *Tac* tacrolimus, *CsA* cyclosporine, *MPA* mycophenolic acid, *MMF* mycophenolate mofetil, *AZA* azathioprine, C_0_ – trough level

^a^ indicates that only one patient received cyclosporine in this group

Throughout this study, Tregs refer to cells with the combination CD4^+^ CD25^+^ CD127^−^ Foxp3^+^, Helios^+^ Tregs to cells with the combination CD4^+^CD25^+^CD127^−^Foxp3^+^Helios^+^, IFNg^+^ Tregs to CD4^+^CD25^+^CD127^−^Foxp3^+^IFNg^+^, and CTLA-4^+^ Tregs to CD4^+^ CD25^+^ CD127^−^ Foxp3^+^ CD152^+^ (intracellular).

### Vitamin D treatment regimen

Cholecalciferol or calcitriol was supplemented in different regimens and dosages. Oral calcitriol (Rocaltrol®) was prescribed on a daily basis in 83 %, on alternate days in 11 %, on a weekly basis in 4 %, and on 5 consecutive days per week in 2 % of cases. Oral cholecalciferol (Ideos®, Dekristol®, Calcimagon®, or Vigantoletten®) was prescribed in a once-weekly dose in 70 %, and on a daily basis in 30 % of the cases. The average prescribed dosages are shown in Table 1.

### Determination of PBL subsets

PBL subsets were determined as described previously [24, 25]. For analysis of cell surface determinants, PBL were incubated with fluorochrome-labelled monoclonal antibodies against CD4 (clone RPA-T4), CD25 (clone M - A251), CD127 (clone HIL-7R-M21) (all from BD Biosciences). Intracellular determinants were stained with fluorochrome-labelled monoclonal antibodies against Foxp3 (clone 236A/E7), IFNg (clone B27), CD152 (BN13) and Helios (clone 22 F6) (all BD Biosciences). Briefly, PBL were incubated with combinations of monoclonal antibodies for 30 min and eight-color fluorescence was analyzed using a FACSCanto II triple-laser flow cytometer (BD Biosciences) [24, 25]. When, in addition, intracellular proteins were studied, cell membranes were permeabilized using BD Perm/Wash buffer (BD Biosciences). At least 100,000 events were analyzed in the initial FSC/SSC dot plot.

### Statistical analysis

Data are presented as median + interquartile range or percentages. Kruskal-Wallis, Fisher’s exact, and Spearman’s rank tests were applied. Bonferroni correction for multiple comparisons was performed when indicated.

## Results

Table 1 shows the characteristics of transplanted patients and healthy controls. Although the healthy control group was significantly different from the patient groups regarding age and sex, this did not impact our results, as we did not find a correlation between age or gender and the numbers of Helios^+^, Helios^−^, CTLA-4^+^, IFNg^+^, and total Tregs in the patients (*p* = NS).

### Controls vs. transplanted patients not supplemented with vitamin D

**Fig. 1:**
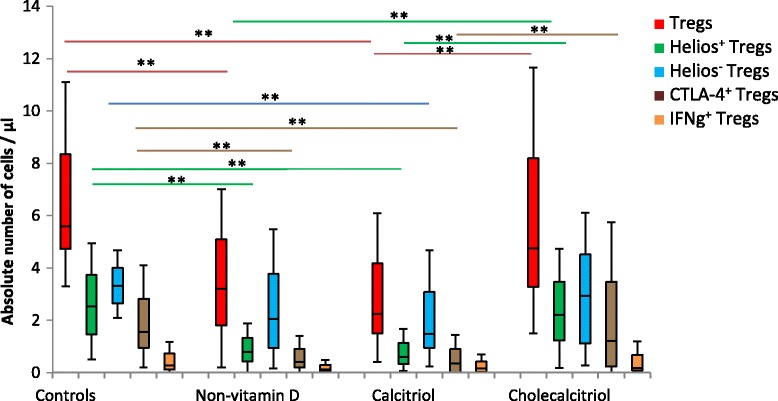
Absolute numbers of Treg subsets among the different study groups. Asterisks in the diagram indicate adjusted *p* values of ≤ 0.005. The exact *p* values for the pairwise comparisons are shown in Table 2

Healthy controls showed higher counts than the transplanted patients who were not supplemented with any vitamin D form regarding the numbers of Helios^+^ (CD4^+^CD25^+^CD127^−^Foxp3^+^Helios^+^), CTLA-4^+^ (CD4^+^CD25^+^CD127^−^Foxp3^+^CD152^+^), and total Tregs (CD4^+^CD25^+^CD127^−^Foxp3^+^) (Table 2, Fig. 1). The numbers of Helios^−^ Tregs (CD4^+^CD25^+^CD127^−^Foxp3^+^Helios^-^) were also higher in the control group, although the difference did not remain significant after correcting for multiple comparisons. IFNg^+^ Treg (CD4^+^CD25^+^CD127^−^Foxp3^+^IFNg^+^) numbers were similar in the control and non-vitamin D groups. We conclude that post-transplant immunosuppressed patients have lower numbers of most Treg subsets than healthy controls.

**Table 2:** Pairwise comparisons of *p*- values of Helios^+^, Helios^−^, CTLA-4^+^, IFNg^+^, and Treg differences, in addition to Treg subset ratios among non-vitamin D, calcitriol and cholecalciferol groups based on Kruskal-Wallis test (*n* = 139)

	Controls	Non-vitamin D	Calcitriol	Cholecalciferol	*p* value
No. of subjects	16	69	38	16	
Total Tregs*		√	√		0.108
			√	√	**0.001**
	√		√		**<0.001**
		√		√	0.016
	√	√			**<0.001**
	√			√	0.326
Helios^+^ Tregs*		√	√		0.353
			√	√	**<0.001**
	√		√		**<0.001**
		√		√	**0.001**
	√	√			**<0.001**
	√			√	0.548
Helios^−^ Tregs*		√	√		0.101
			√	√	0.062
	√		√		**0.001**
		√		√	0.418
	√	√			0.011
	√			√	0.173
CTLA-4^+^ Tregs*		√	√		0.268
			√	√	**0.004**
	√		√		**<0.001**
		√		√	0.022
	√	√			**<0.001**
	√			√	0.207
IFNg^+^ Tregs*		√	√		0.535
			√	√	0.024
	√		√		0.016
		√		√	0.095
	√	√			0.069
	√			√	0.898
Treg/CD4^+^ T cells		√	√		0.872
			√	√	**0.002**
	√		√		**0.004**
		√		√	**0.002**
	√	√			**0.003**
	√			√	0.861
Treg/CD8^+^ T cells		√	√		0.177
			√	√	**0.008**
	√		√		**0.001**
		√		√	0.062
	√	√			0.009
	√			√	0.599
Helios^+^/Helios^−^ Tregs		√	√		0.687
			√	√	0.018
	√		√		0.014
		√		√	0.024
	√	√			0.019
	√			√	0.941

Pairwise comparisons among the four groups. *p*-values ≤0.008 were considered significant due to Bonferroni correction of α- values for multiple comparisons. Significant *p*-values are bold printed. Asterisks refer to Tregs with the marker combination of CD4^+^ CD25^+^ CD127^−^ Foxp3^+^

### Vitamin D effect on Treg subsets

- Calcitriol vs. Controls
  Healthy controls had higher numbers of Helios^+^ (CD4^+^CD25^+^CD127^−^Foxp3^+^Helios^+^), Helios^−^ (CD4^+^CD25^+^CD127^−^Foxp3^+^Helios^-^), CTLA-4^+^ (CD4^+^CD25^+^CD127^−^Foxp3^+^CD152^+^) and total Tregs (CD4^+^CD25^+^CD127^−^Foxp3^+^) than calcitriol-treated patients (Table 2, Fig. 1). The numbers of IFNg^+^ Tregs (CD4^+^CD25^+^CD127^−^Foxp3^+^IFNg^+^) were similar in the two groups.
- Calcitriol vs. no vitamin D supplementation
  Both groups showed similar numbers of Helios^+^ (CD4^+^CD25^+^CD127^−^Foxp3^+^Helios^+^), Helios^−^ (CD4^+^CD25^+^CD127^−^Foxp3^+^Helios^−^), CTLA-4^+^ (CD4^+^CD25^+^CD127^−^Foxp3^+^CD152^+^), IFNg^+^ (CD4^+^CD25^+^CD127^−^Foxp3^+^IFNg^+^), and total Tregs (CD4^+^CD25^+^CD127^−^Foxp3^+^) (Table 2, Fig. 1).
- Cholecalciferol supplementation vs. Controls
  Interestingly, cholecalciferol-treated patients showed similar numbers of Helios^+^ (CD4^+^CD25^+^CD127^−^Foxp3^+^Helios^+^), Helios^−^ (CD4^+^CD25^+^CD127^−^Foxp3^+^Helios^−^), CTLA-4^+^ (CD4^+^CD25^+^CD127^−^Foxp3^+^CD152^+^), IFNg^+^ (CD4^+^CD25^+^CD127^−^Foxp3^+^IFNg^+^), and total Tregs (CD4^+^CD25^+^CD127^−^Foxp3^+^) compared to the control group (Table 2, Fig. 1).
- Cholecalciferol vs. no vitamin D supplementation
  Cholecalciferol supplementation was associated with higher numbers of Helios^+^ (CD4^+^CD25^+^CD127^−^Foxp3^+^Helios^+^), CTLA-4^+^ (CD4^+^CD25^+^CD127^−^Foxp3^+^CD152^+^) and total Tregs (CD4^+^CD25^+^CD127^−^Foxp3^+^), although the difference regarding the latter two variables did not remain significant after correcting for multiple comparisons (Table 2, Fig. 1). The numbers of IFNg^+^ (CD4^+^CD25^+^CD127^−^Foxp3^+^IFNg^+^) and Helios^−^ Tregs (CD4^+^CD25^+^CD127^−^Foxp3^+^Helios^−^) were similar in the cholecalciferol and non-vitamin D groups. We conclude that cholecalciferol is superior to no treatment with vitamin D supplementation regarding the achievement of higher numbers of Helios^+^ Tregs (CD4^+^CD25^+^CD127^−^Foxp3^+^Helios^+^).
- Calcitriol vs. Cholecalciferol supplementation
  Cholecalciferol was associated with two-fold higher numbers of Helios^+^ (CD4^+^CD25^+^CD127^−^Foxp3^+^Helios^+^) and total Tregs (CD4^+^CD25^+^CD127^−^Foxp3^+^) than calcitriol. CTLA-4^+^ Treg (CD4^+^CD25^+^CD127^−^Foxp3^+^CD152^+^) numbers were also higher in the cholecalciferol than in the calcitriol group (Table 2, Fig. 1). We conclude that cholecalciferol is superior to calcitriol with respect to the numbers of Helios^+^, CTLA-4^+^, and total Tregs.

Based on these comparisons, we conclude that cholecalciferol appears to have a stabilizing effect on Tregs, particularly the tTreg subset, since the patients in the cholecalciferol group had similar numbers of Helios^+^ and total Tregs to healthy controls, and higher Helios^+^ and total Treg numbers than the calcitriol supplemented and non-vitamin D patients.

### Treg/CD4+ T cell ratio

When Treg subsets (CD4^+^CD25^+^CD127^−^Foxp3^+^) were analyzed with respect to total CD4, cholecalciferol-treated patients showed similar Treg subset percentages as healthy controls, but higher than calcitriol and non-vitamin D patients. The result suggests that the proportion of the Treg subset with respect to the total CD4^+^ pool was normal in cholecalciferol-treated patients but decreased in calcitriol and non-vitamin D patients, implying a stabilization of Treg subsets by supplementation with cholecalciferol (Tables 1 and 2).

### Helios^+^/Helios^−^ Tregs

Cholecalciferol-treated patients and healthy controls showed a relative dominance of Helios^+^ Treg (CD4^+^CD25^+^CD127^−^Foxp3^+^Helios^+^), whereas calcitriol and non-vitamin D patients exhibited a preponderance of Helios^−^ Treg subsets (CD4^+^CD25^+^CD127^−^Foxp3^+^Helios^-^) (Tables 1 and 2). It appears that Helios^+^ as well as Helios^−^ Treg absolute counts were reduced in calcitriol and non-vitamin D patients but were stable in patients supplemented with cholecalciferol. Moreover, the reduction of Helios^+^ Treg appeared to be stronger than the reduction of Helios^−^ Treg.

### CD8^+^ T, CD16^+^CD56^+^ NK, and CD19^+^ B cells

There was a tendency of cholecalciferol-treated patients to have high CD8^+^ cytotoxic T-lymphocyte and CD16^+^CD56^+^ natural killer cell counts in the absence of clinical symptoms or indications of infection, suggesting high immunocompetence of cholecalciferol-treated patients (Tables 1 and 2). All immunosuppressed patients had extremely low B-lymphocyte counts.

### Higher-dose calcitriol supplementation

We also analyzed whether higher doses of calcitriol (daily doses ≥ 0.5 μg) were associated with higher numbers of Helios^+^ (CD4^+^CD25^+^CD127^−^Foxp3^+^Helios^+^), Helios^−^ (CD4^+^CD25^+^CD127^−^Foxp3^+^Helios^−^), CTLA-4^+^ (CD4^+^CD25^+^CD127^−^Foxp3^+^CD152^+^), and total Tregs (CD4^+^CD25^+^CD127^−^Foxp3^+^) than lower doses, perhaps comparable to those in the cholecalciferol and control groups of transplanted patients (Table 3). Forty-two percent of the transplanted patients in the calcitriol arm received relatively high doses of calcitriol supplementation at an average weekly dose of 3.5 μg (3.5–7 μg). This higher-dose calcitriol group was similar to the cholecalciferol and control groups regarding age, sex, follow up time post transplantation, the percentage that received anti-thymocyte globulin induction, and maintenance immunosuppressive protocols (*p* = NS) (Data not shown).

**Table 3:** CD4^+^ and Treg subset counts and ratios of Helios^+^ and total Treg cell counts in the higher-dose calcitriol group vs. cholecalciferol and non-vitamin D groups (*n* = 101)

	Non-vitamin D	Higher-dose Calcitriol	Cholecalciferol	*P* value
No. of subjects	69	16	16	
CD4^+^ T cells/μl	677 (373–1043)	458 (315–879)	595 (352–1070)	0.544
Tregs/μl*	3.1 (1.6–5.3)	2.3 (1.2–4.1)	4.7 (3–8.5)	0.021
Helios^+^ Tregs/μl*	0.7 (0.3–1.4)	0.4 (0.2–1.6)	2.2 (1.1–3.8)	0.001
Helios^−^ Tregs/μl*	2.2 (0.9–3.9)	1.4 (0.9–3)	2.7 (1.1–4.6)	0.393
IFNg^+^ Tregs/μl*	0.3 (.04–0.3)	0.16 (0.06–0.4)	0.2 (0.1–0.7)	0.06
CTLA-4^+^ Tregs/μl*	0.4 (0.2–0.9)	0.25 (0–0.7)	1.2 (0.2–3.5)	0.018
Treg/CD4^+^ T cells %	0.45 (0.29–0.78)	0.44 (0.25–0.95)	0.84 (0.53–1.4)	0.006
Treg/ CD8^+^ T cells %	0.84 (0.37–1.5)	0.57 (0.27–1.2)	1.4 (0.9–1.8)	0.058
Helios^+^/ Helios^−^ Tregs	0.38 (0.27–0.61)	0.28 (0.19–0.97)	0.65 (0.38–1.4)	0.048

Tregs denoted with asterisks have the marker combination CD4^+^ CD25^+^ CD127^−^ Foxp3^+^

- Higher-dose calcitriol vs. no vitamin D supplementation
  Even at higher doses, calcitriol was not superior to no vitamin D supplementation with respect to the numbers of Helios^+^ (CD4^+^CD25^+^CD127^−^Foxp3^+^Helios^+^), CTLA-4^+^ (CD4^+^CD25^+^CD127^−^Foxp3^+^CD152^+^), and total Tregs (CD4^+^CD25^+^CD127^−^Foxp3^+^). The numbers of IFNg^+^ (CD4^+^CD25^+^CD127^−^Foxp3^+^IFNg^+^) and Helios^−^ Tregs (CD4^+^CD25^+^CD127^−^Foxp3^+^Helios^−^) likewise were similar in the two groups (Tables 3 and 4).

**Table 4:** Pairwise comparisons of *p*-values of Helios^+^, CTLA-4, and Treg differences, in addition to Treg subset ratios among non-vitamin D, calcitriol and cholecalciferol groups based on Kruskal-Wallis test (*n* = 101)

	Non-vitamin D	Higher-dose Calcitriol	Cholecalciferol	P value
No. of subjects	69	16	16	
Total Tregs*	√	√		0.349
		√	√	**0.009**
	√		√	**0.016**
Helios^+^ Tregs*	√	√		0.553
		√	√	**0.002**
	√		√	**0.001**
CTLA-4^+^ Tregs*	√	√		0.152
		√	√	**0.005**
	√		√	0.034
Treg/CD4^+^ T cells %	√	√		0.951
		√	√	**0.012**
	√		√	**0.002**
Helios^+^/ Helios^−^ Tregs	√	√		0.507
		√	√	0.024
	√		√	0.027

*P* values ≤ 0.016 were considered significant due to Bonferroni correction of α-values for multiple comparisons. Significant *p*-values are bold printed. Asterisks refer to Tregs with the marker combination CD4^+^ CD25^+^ CD127^−^ Foxp3^+^
- Higher-dose calcitriol vs. Cholecalciferol supplementation
  The absolute numbers of Helios^+^, CTLA-4^+^ (CD4^+^CD25^+^CD127^−^Foxp3^+^CD152^+^), and total Tregs (CD4^+^CD25^+^CD127^−^Foxp3^+^) were higher in the cholecalciferol group than in the calcitriol group. The numbers of IFNg^+^ (CD4^+^CD25^+^CD127^−^Foxp3^+^IFNg^+^) and Helios^−^ Tregs (CD4^+^CD25^+^CD127^−^Foxp3^+^Helios^−^) were similar in the two groups (Tables 3 and 4).

### Correlation between Helios^+^ and CTLA-4^+^ Tregs

We also tested whether there was a correlation between the absolute numbers of Helios^+^ Tregs (CD4^+^CD25^+^CD127^−^Foxp3^+^Helios^+^) and the number of CTLA-4^+^ Tregs (CD4^+^CD25^+^CD127^−^Foxp3^+^CD152^+^). We observed a moderate correlation in the cholecalciferol group (*r* = 0.670, *p* = 0.003), and the calcitriol group (*r* = 0.470, *p* = 0.001), and a weak correlation in the non-vitamin D group (*r* = 0.301, *p* =0.012). The maximum correlation was observed in healthy controls (*r* = 0.776, *p* < 0.001).

### Serum creatinine

There was no significant difference in serum creatinine (mg/dl) among the patient groups (*p* = 0.155).

## Discussion

In this retrospective study of renal allograft recipients, we tested a possible association between vitamin D supplementation with either cholecalciferol or calcitriol and the numbers of Tregs. We found that supplementation with cholecalciferol was associated with higher absolute numbers of Helios^+^ Tregs (CD4^+^CD25^+^CD127^−^Foxp3^+^Helios^+^) than calcitriol or no vitamin D supplementation, and with higher absolute numbers of total Tregs (CD4^+^CD25^+^CD127^−^Foxp3^+^) than with calcitriol supplementation. The absolute numbers of Treg subsets were similar in the cholecalciferol group to healthy controls. We did not find a significant difference in the numbers of Helios^−^ (CD4^+^CD25^+^CD127^−^Foxp3^+^Helios^−^) and IFNg^+^ (CD4^+^CD25^+^CD127^−^Foxp3^+^IFNg^+^) Tregs among the 3 renal allograft patient groups.

We assessed absolute number of cells/μl in addition to the ratio of Tregs to CD4^+^ and CD8^+^ cells. Liu et al. demonstrated that the absolute numbers of Tregs, rather than the ratios of Tregs to peripheral lymphocytes, was associated with long-term survival of renal allografts [26].

Interestingly, as shown in Table 1, in our study cholecalciferol at an average dose of 7000 IU weekly was associated with higher numbers of Helios^+^ and total Tregs than calcitriol at an average dose of 1.75 μg weekly. In vivo trials reported cholecalciferol supplementation at doses of 140,000 IU monthly or 20,000 IU daily, and 0.5 μg of daily calcitriol in another study [17, 20, 21, 27]. The average dose of cholecalciferol supplemented in our study was thus about 5–20 times lower than the dosages prescribed in these trials, whereas the average dose of calcitriol was about 3 times lower than that administered in a trial conducted by Ardalan et al. [17]. Our results show that cholecalciferol has an effect on Tregs in transplant patients even at relatively low doses.

Although calcitriol can directly affect T cells in vitro, the required doses are much higher than the physiological doses as demonstrated in many studies [16, 28–31]. In addition, it was demonstrated in healthy persons as well as uremic patients that the bioavailability of 1, 25(OH)_2_ D_3_ was 70 % of the supplemented dose of oral calcitriol [32]. De Sévaux et al. showed that calcitriol supplementation in a daily dose of 0.25 μg did not improve the serum 25(OH) D_3_ level in renal transplant patients [33]. Consistent with this finding, Marcen et al. demonstrated that calcitriol in a daily dose of 0.25–0.5 μg failed to improve vitamin D deficiency in a cohort of renal transplant patients [34]. In contrast, low dose oral cholecalciferol in a weekly dose of 5000 IU for 15 weeks increased serum 25(OH) D_3_ from 18.4 ± 8.2 to 68.6 ± 17.7 nmol/l in a cohort of 34 hemodialysis patients without causing any episode of hypercalcemia [35]. Moreover, oral cholecalciferol showed good long-term 25(OH) D_3_ and 1, 25 (OH)_2_ D_3_ bioavailability, even after 3 months of supplementation with a single dose of 600,000 IU, with a maximal effect at one month [36]. Based on these findings, we speculate that the calcitriol doses supplemented in our study were much lower than the doses required for induction of Tregs in patients, in contrast to cholecalciferol, which can improve vitamin D deficiency even at low doses.

Our study challenges the findings of Ardalan et al., who reported that calcitriol administered to donors 5 days before transplantation at a dose of 0.5 μg and continued at the same dose in the recipients for 1 month after transplantation and thereafter at a dose of 0.25 μg for another 5 months, was associated with a significant increase in the numbers of CD4^+^CD25^+^ cells [17]. This finding is surprising since the doses administered seem too low for the induction of a significant increase of Tregs in the light of other studies. The most likely explanation of the discrepancy is that the Treg cell population of our study was defined as CD4^+^ CD25^+^ Foxp3^+^ CD127 ^low/-^, whereas in Ardalan’s trial Tregs were defined only as CD3^+^CD4^+^CD25^+^ T cells. It was shown that a subpopulation of Tregs, termed IL-10-Tregs, expressed CD4 and CD25, whereas these cells lacked the expression of Foxp3 [37]. The marker combination CD4^+^ CD25^+^ IL10^+^ characterizes a Treg subset termed Tr1 cells. Because only the CD4^+^ T cell subset, which expresses the highest level of CD25 (CD25 ^high^), has a suppressive effect, it is likely that the cell population studied by Ardalan et al. was a mixture of Tr1 cells in addition to both CD4^+^ CD25^+^ effector cells and conventional Tregs [38, 39]. Moreover, serum 1, 25 (OH)_2_ D_3_ was not measured before and six months after transplantation. This would have provided an idea about the effects of the supplemented doses on serum 1, 25 (OH)_2_ D_3_. To test whether the higher doses of calcitriol administered in Ardalan’s study were responsible for the increase in the numbers of Tregs, we compared the patients who were supplemented with comparable or higher doses of calcitriol as the patients in Ardalan’s trial with the other two groups of transplanted patients in our study. The higher- dose calcitriol group received an average dose of 3.5 μg weekly. We found that the Tregs were still about two-folds higher in the cholecalciferol arm. This finding suggests that cholecalciferol is superior to calcitriol even when the latter is prescribed at higher doses.

Although cholecalciferol was associated with higher numbers of Helios^+^ Tregs (CD4^+^CD25^+^CD127^−^Foxp3^+^Helios^+^), we could not detect a significant difference in the numbers of Helios^−^ Tregs (CD4^+^CD25^+^CD127^−^Foxp3^+^Helios^-^) among cholecalciferol, calcitriol, no vitamin D supplementation, and control groups. Since Helios^−^ Tregs represent a mixture of tTregs (Bona fide Tregs) and a majority of Tregs activated when exposed to antigens (pTregs) [4], we speculate that cholecalciferol might have affected only the bona fide tTregs rather than peripherally activated Tregs. We think that even if cholecalciferol caused an increase in the numbers of bona fide Helios^−^ Tregs, the increase might have been too small to be statistically significant, considering that the majority of Helios^−^ Tregs were pTregs and that the sample size was small. If this hypothesis proves to be true, the important question is how such relatively low doses of cholecalciferol can affect the bona fide Tregs (either Helios^+^ or Helios^−^) while not affecting the Helios^−^ pTregs. This may be attributed to the vitamin D-binding protein (DBP). A likely explanation is that the Helios^−^ bona fide tTregs are induced in the thymus where DBP concentrations are much lower than in the serum [40, 41]. The lower concentration of DBP in the thymus renders relatively lower concentrations of cholecalciferol capable of inducing Helios^−^ bona fide tTregs. As Helios^−^ pTregs are induced in the periphery, where the concentrations of DBP are much higher, higher concentrations of 25 (OH) D_3_ may be required to induce pTregs. T cells express CYP27B1, which is a 1α-hydroxylase, and have the capacity to convert 25(OH) D_3_ to 1, 25(OH)_2_ D_3_ in sufficient concentrations to affect the vitamin D-responsive genes [22].

To find out whether the increase in Treg subset numbers associated with cholecalciferol supplementation was real, we compared cholecalciferol patients with healthy controls. Interestingly, both cholecalciferol groups showed similar Treg subset numbers, whereas calcitriol and no vitamin D supplementation groups showed lower Treg numbers. Accordingly, it appears that cholecalciferol has a stabilizing effect on Treg (particularly Helios^+^ subset) rather than a proliferation-inducing one.

CTLA-4 is a marker associated with the suppressive capacity of Tregs. Cholecalciferol was associated with significantly higher numbers of these cells in comparison to calcitriol and there was a trend of higher numbers also in the no vitamin D supplementation group, whereas the numbers were comparable to healthy controls. We tested the correlation between CTLA-4^+^ Tregs (CD4^+^CD25^+^CD127^−^Foxp3^+^CD152^+^) and Helios^+^ Tregs (CD4^+^CD25^+^CD127^−^Foxp3^+^Helios^+^) in the four groups to estimate whether Helios^+^ Tregs co-express CTLA-4 and use CTLA-4 for cell-cell contact suppression. Helios^+^ Tregs were associated with CTLA-4 in all 4 groups suggesting that they use CTLA-4 for suppression.

Immunosuppressive drugs have variable effects on Tregs. Cyclosporine has long been known to inhibit the activation of T cells through suppression of calcium-dependent phosphatase calcineurin leading to suppression of IL-2 synthesis. Melony et al. showed that calcineurin inhibitors (CNI) led to expansion of the Treg population in lung allograft recipients [42]. Intriguingly, Ruppert et al. showed that Tregs resisted apoptosis caused by cyclosporine through expression of CD44 [43]. Kogina et al. reported that tacrolimus suppressed T cell receptor-mediated cell division of conventional T cells (CD4^+^ T cells), whereas it enhanced division of Tregs in vitro [44]. In contrast, other studies showed a harmful effect of CNI on Tregs [45, 46]. In our study, it is unlikely that calcineurin inhibitors were the cause of the increased Treg numbers in the cholecalciferol group as we found no significant difference in CNI prescription among the three patient groups. Mycophenolic acid is one of the most widely used drugs in solid organ transplantation. It exerts its function through inhibition of inosine monophosphate dehydrogenase leading ultimately to B and T cell suppression. Recently, Scotta et al. showed that administration of methylprednisolone, tacrolimus and mycophenolic acid suppressed the viability and proliferation of Tregs. They have also showed that in vivo administration of sirolimus; an inhibitor of the mechanistic target of rapamycin, maintained proliferation and survival of adoptively transferred Tregs [47]. A recent in vitro study published by our group showed variable effects of the immunosuppressive agents on IFNg^+^ and total Tregs [48].

## Conclusions

It appears that cholecalciferol prevents the decrease of Treg commonly observed after transplantation and believed to be a consequence of immunosuppression. Particularly the decrease of Helios^+^ tTregs (CD4^+^CD25^+^CD127^−^Foxp3^+^Helios^+^), which usually inhibit autoreactive T effector cells and thus guard against autoimmune diseases, appears to be prevented. Moreover, Helios^−^ pTregs (CD4^+^CD25^+^CD127^−^Foxp3^+^Helios^−^) are stabilized in cholecalciferol-treated patients. It might be permissible to speculate that cholecalciferol-treated patients with high Treg numbers may require lower doses of immunosuppressive drugs, thereby reducing the side-effects and cost of immunosuppression.

If vitamin D deficiency proves to be the cause of Treg deficiency in immunosuppressed patients, and calcitriol cannot improve this deficiency, cholecalciferol, even when administered at relatively low doses may be able to reverse vitamin D deficiency and thereby restore Treg balance.

Since approximately 51 % of transplant recipients have a vitamin D insufficiency and about 29 % have moderate to severe vitamin D deficiency and patients with low dose cholecalciferol supplementation rarely develop hypercalcemia [49], the majority of transplant recipients should be supplemented with cholecalciferol. Further clinical studies will be necessary to validate our hypothesis and conclusion.

Since our study is cross-sectional, we can only infer an association between cholecalciferol and increased numbers of Helios^+^, CTLA-4 ^+^, and total Tregs rather than causality. To prove causality, a randomized prospective study should be conducted. Most of the renal transplant patients in the centers from which the patients were recruited are managed with calcitriol supplementation rather than cholecalciferol. Therefore, we could not enroll more patients into the latter group.

### Ethics approval and consent to participate

The study was reviewed by the ethics committee of Heidelberg University and was carried out in accordance with the ethical standards laid down in the 2000 declaration of Helsinki as well as the declaration of Istanbul 2008. All participants gave informed consent prior to their inclusion in the study.

### Availability of data and materials

Unfortunately, our raw data will not be shared because they can reveal the identity of the participants. The healthy controls did not accept sharing their data. In addition, the raw data are currently being analyzed in other projects.

## Abbreviations

CNI: calcineurin inhibitors
CTLA-4: cytotoxic T-lymphocyte-associated protein 4
DBP: vitamin D-binding protein
Foxp3: forkhead box P3
IFNg: interferon- gamma
iTregs: in vitro-induced regulatory T-cells
NK: natural killer cells
PBL: peripheral blood lymphocytes
pTregs: peripheral regulatory T-cells
Tregs: regulatory T-cells
tTregs: thymic regulatory T-cells
VDR: vitamin D- receptor
VDRE: vitamin D-response elements
